# Revitalising Riboflavin: Unveiling Its Timeless Significance in Human Physiology and Health

**DOI:** 10.3390/foods13142255

**Published:** 2024-07-17

**Authors:** M. Ângela Aragão, Lara Pires, Celestino Santos-Buelga, Lillian Barros, Ricardo C. Calhelha

**Affiliations:** 1 Centro de Investigação de Montanha (CIMO), Instituto Politécnico de Bragança, Campus de Santa Apolónia, 5300-253 Bragança, Portugal; angelaragao@ipb.pt (M.Â.A.); laravaqueiro@ipb.pt (L.P.); lillian@ipb.pt (L.B.); 2 Laboratório Associado para Sustentabilidade e Tecnologia em Regiões de Montanha (SusTEC), Instituto Politécnico de Bragança, Campus de Santa Apolónia, 5300-253 Bragança, Portugal; 3 Grupo de Investigación en Polifenoles (GIP-USAL), Facultad de Farmacia, Campus Miguel de Unamuno, Universidad de Salamanca, s/n, 37007 Salamanca, Spain; csb@usal.es

**Keywords:** riboflavin, human health, coenzyme, metabolic function, therapeutical potential

## Abstract

Since the early twentieth century, research on vitamins has revealed their therapeutic potential beyond their role as essential micronutrients. Riboflavin, known as vitamin B2, stands out for its unique characteristics. Despite numerous studies, riboflavin remains vital, with implications for human health. Abundantly present in various foods, riboflavin acts as a coenzyme in numerous enzymatic reactions crucial for human metabolism. Its role in energy production, erythrocyte synthesis, and vitamin metabolism underscores its importance in maintaining homeostasis. The impact of riboflavin extends to neurological function, skin health, and cardiovascular well-being, with adequate levels linked to reduced risks of various ailments. However, inadequate intake or physiological stress can lead to deficiency, a condition that poses serious health risks, including severe complications. This underscores the importance of maintaining sufficient levels of riboflavin for general wellness. The essential role of riboflavin in immune function further emphasises its significance for human health and vitality. This paper examines the diverse effects of riboflavin on health and stresses the importance of maintaining sufficient levels for overall well-being.

## 1. Introduction

Over the past century, extensive research into the biological, chemical, and physical properties of vitamins has revealed their therapeutic benefits beyond their fundamental role as essential micronutrients for human health and development [1].

Riboflavin, referred to as vitamin B2 (vB2), is a water-soluble vitamin that belongs to the B complex, with an important role in the metabolism of amino acids. Its structure is closely associated with flavins, a significant category of natural dyes in plants and animals. Before researchers discovered its vitaminic properties, the British chemist Alexander Wynter Blyth observed the presence in milk of a yellow-green fluorescent pigment in the 1870s, which was later found in eggs, liver, beer yeast, and fruits, resulting in various names such as lactoflavin, ovoflavin, hepatoflavin, among others [2]. It was not until the early 1930s that Paul Karrer from Zurich, Switzerland, and Richard Kuhn from Heidelberg, Germany, successfully determined the chemical structure of the substance, named riboflavin due to its yellow colour (flavus in Latin), consisting of a flavin ring system, derived from isoalloxazine (i.e., benzo[g]pteridine-2,4(1H,3H)-dione), bearing a ribitol [3]. 

In the 1940s, the first studies on the effects of riboflavin deficiency in humans were reported, and, at the end of the 1960s, the glutathione reductase activity test was proposed to measure riboflavin levels, which continues to be utilised in modern times [4]. Riboflavin (Supplementary Materials) has been a subject of interest for researchers, particularly during the 1960s to 1970s, because of its function as a precursor of flavoenzymes and their biochemical activities. The quantity of publications on this subject remained fairly consistent until the early 2000s, when the emergence of nanotechnology triggered a notable increase in research output. This growth, particularly in health technologies and bioengineering, underscores the growing importance of riboflavin in these areas, making its study and understanding crucial for future advancements. 

Researchers find it a fascinating topic due to its numerous crucial roles in human physiology [5]. It is a water-soluble vitamin not stored in the body, which excretes any excess through urine, and serves as a precursor to two major coenzymes, flavin mononucleotide (FMN) and flavin adenine dinucleotide (FAD), that act as essential cofactors in numerous enzymatic reactions across all life forms [6,7]. The conversion of riboflavin to its coenzyme forms mostly happens in the cytoplasm (the capacity for this conversion can vary depending on the cell type and its metabolic needs), with some evidence suggesting that conversion to FAD may also occur in the mitochondria and nucleus. First, the enzyme riboflavin kinase catalyses riboflavin ATP-dependent phosphorylation in the presence of Zn^2+^; the result of this process is FMN [8]. Only a tiny portion of FMN is directly used as a coenzyme, whereas the majority undergoes modification by adding a pyrophosphate-bridged adenyl moiety. This modification occurs through a process involving Mg^2+^ and the enzyme FAD synthetase, producing FAD. The FAD content that is present in tissues controls the conversion process. It has been shown in rats that an excessive amount of FAD hinders this conversion [9].

Certain enzymes chemically bond a portion of the produced FAD, playing crucial roles in metabolism [10]. The majority of flavoproteins include a firmly but noncovalently attached flavin molecule, while only around 10% of all flavoproteins have a covalently attached flavin molecule. Examples of the latter include sarcosine dehydrogenase, succinic dehydrogenase, and monoamine oxidases A and B. Thyroid hormones actively regulate the process of synthesising flavin coenzymes and forming covalent bonds with flavins. Around 84% of human flavoproteins rely on FAD as a cofactor, whereas only 16% use FMN [11].

Riboflavin is abundantly present in a variety of foods including dairy products like milk and cheese, meats such as beef and chicken, fish like salmon and trout, and a diverse range of fruits and vegetables, including bananas, spinach, and broccoli [12,13]. This wide distribution of riboflavin in our diet reassures us of its accessibility and potential health benefits. 

Riboflavin is a key predominant energy supplier for cells, and a vital component in the electron transport chain, a series of processes that generate ATP. In general, its effects contribute to the maintenance of the body’s homeostasis [14]. Furthermore, it also enhances the metabolic processes involving vitamins such as niacin, pyridoxine, folic acid, and cobalamin by working with FMN and FAD [15]. It also plays an essential role in the synthesis of erythrocytes, which are critical in transporting oxygen throughout the body, and it is crucial for maintaining the neurological system, skin, eyes, and mucous membranes [16]. The concentrations of riboflavin in plasma are inversely correlated with those of homocysteine, an amino acid whose excessive levels are associated with conditions such as cardiovascular diseases, pregnancy problems, and cognitive impairment. [17]. All in all, ensuring sufficient concentrations of riboflavin may aid in preventing or managing many ailments.

Inadequate dietary intake or physiological stress can lead to riboflavin deficiency, a condition that can have serious health implications [18]. These include stunted development, a reduced red blood cell count, skin abnormalities, renal damage, and degenerative changes in the brain, all of which can significantly affect cognitive ability. A more severe riboflavin deficiency can lead to ariboflavinosis [19], a disorder with symptoms like sore throats, swollen mucous membranes, and skin lesions. This underscores the crucial importance of maintaining adequate riboflavin levels.

Riboflavin, as an essential coenzyme in human physiology, plays a pivotal role in numerous metabolic actions that sustain good health and vitality. Its significance extends beyond basic metabolic activities, including its involvement in immune function and protection against micro-organisms, highlighting its fundamental importance for human nutrition and general well-being. [20].

**Figure 1:**
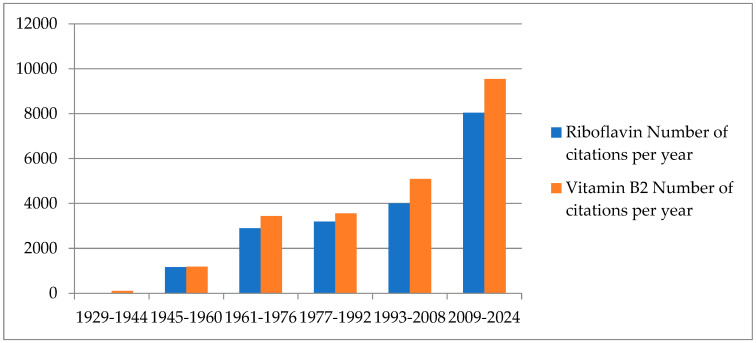
Graph: “Number of publications per year collected in the PubMed database for the terms ‘Riboflavin’ and ‘Vitamin B2’ (search conducted on 9 July 2024)”.

According to the PubMed statistics of publications, since its discovery, interest in studying riboflavin has increased over time (Figure 1), and it continues to be a molecule of extreme interest in several fields.

## 2. Insights into the Biosynthesis of Riboflavin: Microbial and Plant Pathways

While higher animals, including humans, acquire riboflavin from their diet due to the lack of internal production capabilities, certain bacteria, fungi, and plants can synthesise the vitamin independently [21]. Notably, pathogenic Gram-negative enterobacteria, such as *Escherichia coli*, *Mycobacterium tuberculosis*, and *Salmonella typhimurium*, depend significantly on their own production of riboflavin [21]. 

The elucidation of riboflavin biosynthesis began in the early 1950s, motivated by the practical need to produce this vital vitamin for human and animal nutrition [22]. In recent years, fermentation methods using bacteria or yeasts have mostly substituted the chemical production of riboflavin, resulting in an annual output of more than 3000 metric tons [23]. Initial approaches to biological synthesis mostly focused on fungi and yeasts because of their natural ability to produce flavins. *Ascomycetes* like *Eremothecium ashbyii* and *Ashbya gossypii*, along with several species of *Candida* sp., were explored by different researchers [24]. These species were useful models for understanding riboflavin biosynthesis pathways, providing a foundation for further development. However, there has been a recent shift in focus towards reconstructing the pathway in non-flavinogenic bacteria, namely, *E. coli* and *Bacillus subtilis*, as well as the yeast *Saccharomyces cerevisiae*, using them as microbial platforms [25]. This shift is significant as it opens new possibilities for riboflavin production, potentially leading to more efficient and cost-effective methods. 

**Figure 2:**
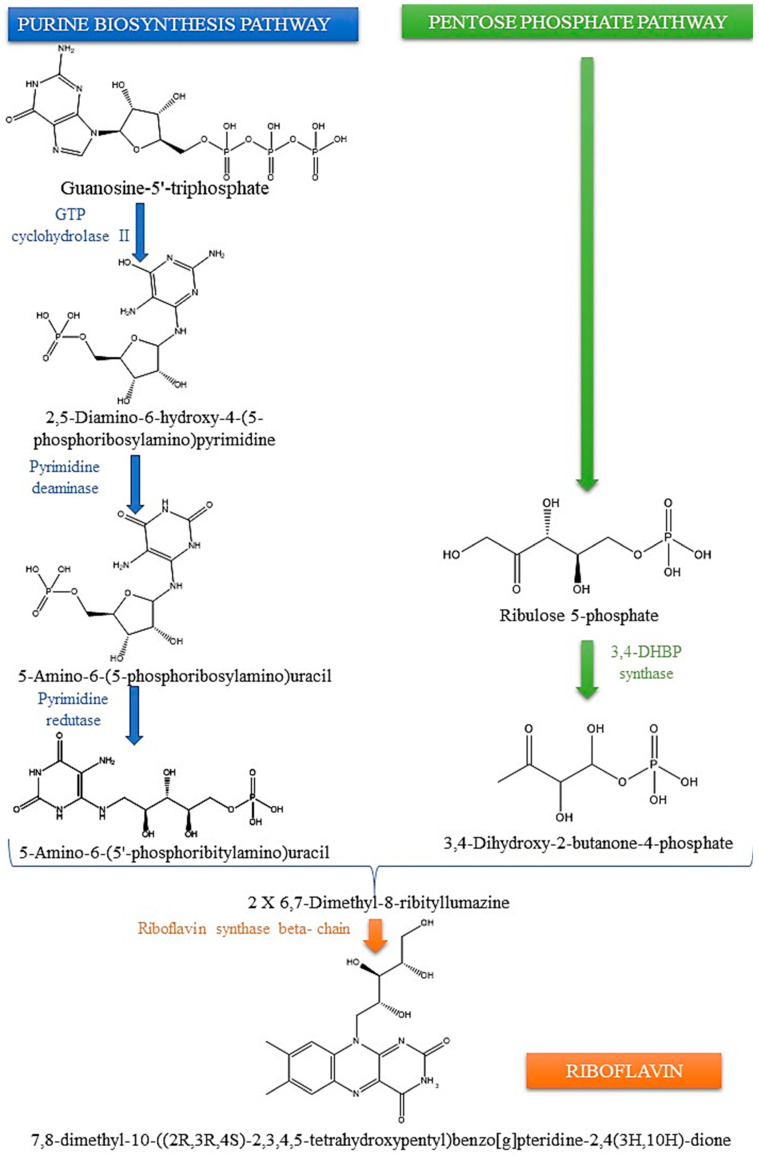
The biosynthesis pathway for riboflavin uses one molecule of GTP from the purine biosynthesis route and two molecules of ribulose-5-phosphate from the pentose phosphate pathway to make one riboflavin molecule. This process occurs via a series of processes catalysed by enzymes. There are two separate parts of the process when 3,4-dihydroxy-2-butanone-4-phosphate and 5-amino-6-(5′-phosphoribitylamino) uracil come together. This reaction results in the formation of the riboflavin precursor, 6,7-dimethyl-8-ribityllumazine.

The production of the essential flavoenzymes FMN and FAD, which play a critical role in several metabolic processes in all living organisms, requires riboflavin (Figure 2). These coenzymes are present in all forms of life and play important roles in many redox reactions, as well as in other processes including DNA repair, light detection, and bioluminescence [26]. As above indicated, plants and several bacteria have the ability to produce riboflavin; therefore, plant-based diets are significant reservoirs of riboflavin for humans, and the riboflavin pathway is a focal point for modifying genetically enhanced crops [27].

**Figure 3:**
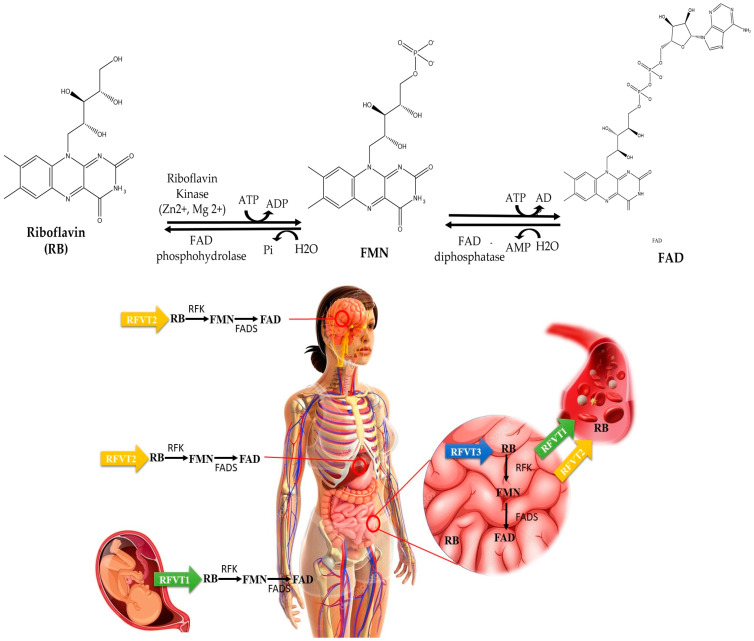
Metabolism and uptake of riboflavin: The gastrointestinal system primarily absorbs riboflavin (RB), with the help of riboflavin transporter 3 (RFVT3). After absorption, gastrointestinal cells can metabolise riboflavin through two pathways. Riboflavin kinase (RFK) can convert it into flavin mononucleotide (FMN), and FAD synthase (FADS) can transform it into flavin adenine dinucleotide (FAD). Alternatively, riboflavin can enter the bloodstream via riboflavin carrier protein-1 (RCP1), transporter 1 (RFVT1), and riboflavin transporter 2 (RFVT2). The circulatory system transports riboflavin to reach its target cells. The maternal circulation transports riboflavin to the foetal bloodstream with the help of RFVT1, expressed in the gastrointestinal system and placenta. RFVT2 is ubiquitously expressed throughout the body and has significant expression in the brain and endocrine organs, including the pancreas. It is also present in the liver and muscle tissue [31]. Riboflavin is used inside the target cells directly or by conversion into either FMN or FAD cofactors for several biological processes. Figure adapted from [32].

The biosynthesis pathway is similar in plants, yeast, and bacteria [28], and involves a series of enzyme-mediated reactions [29] starting from guanosine 5-phosphate (GTP) and two molecules of ribulose 5-phosphate (Ribu5P) (Figure 3) [30]. GTP cyclohydrolase II (also called RibA in *Escherichia coli*) changes GTP into the pyrimidine derivative 2,5-diamino-6-ribosylamino-4(3H)-pyrimidinone 5′-phosphate. This is the first step in the process. Deaminating the pyrimidine ring forms 5-amino-6-ribosylamino-2,4(1H,3H)-pyrimidinedione 5′-phosphate. Afterwards, the ribosyl group is reduced to produce 5-amino-6-ribitylamino-2,4(1H,3H)-pyrimidinedione 5′-phosphate. After the removal of phosphate groups, this substance combines with 3,4-dihydroxy-2-butanone 4-P to yield 6,7-dimethyl-8-ribityllumazine, which can undergo further conversion to form riboflavin [2].

To summarise, the study of riboflavin biosynthesis is a complex and continually advancing field with significant implications for nutrition, biotechnology, and medicine. Progress in understanding riboflavin biosynthesis pathways not only provides crucial insights into fundamental biological processes, but also has the potential to revolutionise treatment strategies for various human health issues.

## 3. The Vital Role of Riboflavin in Human Health

Nutritional riboflavin deficiency occurs when there is an insufficient amount of riboflavin in the diet; this happens because riboflavin is not naturally produced or stored in human tissues, as discussed previously. Hence, it is essential to ensure a sufficient intake of riboflavin throughout the diet. Riboflavin deficiency, which is often asymptomatic, is commonly linked to deficiencies in other vitamins and is typically caused by dietary restrictions [33]. However, it may be more prevalent than currently acknowledged, affecting approximately 10–15% of the world’s population due to an inherited limitation in their ability to absorb or utilise riboflavin [34].

Riboflavin deficiency, also known as ariboflavinosis, is prevalent in underdeveloped nations, particularly in areas where rice is the main dietary staple and there is inadequate consumption of milk and meat [35]. Deficient levels of riboflavin may be triggered or worsened in patients by several conditions, such as alcoholism, diabetes mellitus, liver disease, thyroid and adrenal insufficiency, and gastrointestinal and biliary obstruction [36]. Alcohol consumption hinders the absorption of riboflavin, leading to insufficiency [37]. Additional patient populations more susceptible to riboflavin insufficiency include those with anorexia nervosa, lactose intolerance, or individuals who refrain from consuming milk and dairy products, the primary sources of riboflavin. Medications for seizures and mental health issues, like chlorpromazine, imipramine, and amitriptyline, and some drugs used to treat malaria, like quinacrine, can stop the process of turning riboflavin into its active coenzyme derivatives. Substances, including tetracycline, theophylline, caffeine, and metals like zinc, copper, and iron, tend to chelate or create complexes with riboflavin, which may impact its bioavailability [38]. 

Deficiencies are seldom observable in developing and industrialised nations, where riboflavin is available in a broad range of food. However, while severe instances of ariboflavinosis are rare in those countries, a significant portion of the population experiences a subclinical deficiency phase, characterised by changes in biochemical markers, as can be detected by analysing vitamin levels in bodily fluids, such as blood plasma, urine, and serum [39]. According to the research conducted by Fabian et al. [40], the consumption of vitamins from food was associated with a reasonably low likelihood of inadequate intake in all age and gender categories in European nations. Nevertheless, the authors discovered that, on average, riboflavin consumption was more than 5% lower than the Lower Reference Nutrient Intake (LRNI) [41] and that the deficiency was particularly prominent among young women and the elderly. Flynn et al. [42,43] reported that a significant proportion (60%) of older European individuals could be susceptible to riboflavin deficits. Among demographic groups, besides the elderly, individuals who are alcoholic, are unhealthy, and have kidney problems after dialysis, as well as those with absorption issues, malnourished children, strict vegetarians, athletes, pregnant women, lactating women, infants, and adolescents, are those at particular risk of having a poor riboflavin status in western countries [44]. Ariboflavinosis can be caused not only by improper nutrition but also by the combined effects of certain medications, alcohol consumption, or an increased need due to specific physiological conditions such as pregnancy or breastfeeding, childhood, and old age [45]. Supplementation to avoid ariboflavinosis is often unnecessary as enough riboflavin is obtainable from a well-balanced diet, unless the diet is severely restricted or there are underlying health conditions [46]. 

## 4. Deciphering the Riboflavin Journey: Absorption, Transport, and Metabolism

Despite the fact that higher organisms cannot synthesise riboflavin, bacteria in the large intestine can produce this vitamin internally using ribulose-5-phosphate and GTP. Thus, in humans, riboflavin comes from two origins: food [47], which is absorbed in the small intestine, and bacterial production from the normal microflora of the large intestine, which is absorbed in that part of the gut [48]. It is feasible that a significant portion of riboflavin needs could be met by bacterial production. This source can enhance the overall vitamin burden in the body, particularly by supporting the cellular nutrition and health of colonocytes, the cells lining the colon. These cells are crucial for immune function and nutrient uptake, making riboflavin essential for their role. Riboflavin is present in minimal quantities in food, where it is found in an accessible form, i.e., as an isoalloxazine ring bonded to a ribitol side chain. The main forms of riboflavin in the diet are FMN and FAD [12]. Before absorption, these latter forms undergo hydrolysis by intestinal phosphatases, releasing free riboflavin [49].

As a water-soluble vitamin, the body does not retain it for extended periods; hence, regular intake of the vitamin is necessary. When the amount of riboflavin consumed is around the minimum daily requirement, only 10–20% of the intake is excreted in the urine. A detailed account of how riboflavin is processed in micro-organisms and animals and the production and breakdown of flavoenzymes in both prokaryotic and eukaryotic species can be found in the review by McCormick [50].

The European Food Safety Authority (EFSA) [41] has thoroughly revised the Dietary Reference Values (DRVs) for riboflavin, calculating an estimated average requirement (AR) for adults (>18 years) of 1.3 mg/day. Considering a CV of 10% and rounding to the nearest one decimal place, a Population Reference Intake (PRI) of 1.6 mg/day was established. The EFSA concluded that there is no evidence to suggest that riboflavin needs vary according to sex or age. For infants between 7 to 11 months, where there is insufficient data to determine the average amount of riboflavin required, an intake of 0.4 mg/day is recommended, based on estimates made in exclusively breastfed babies from birth to six months. Allometric scaling is used to adjust the recommendation for children and adolescents aged 1–17 years, according to the differences in body weight, extrapolating downwards from the requirements of adults, considering that riboflavin needs are proportional to the metabolically active body mass. The EFSA also concluded that there is no need to define sex-specific Adequate Intakes (AIs) and PRI for boys and girls of all age groups. Insufficient evidence exists to determine the increased dietary riboflavin requirements for pregnant women so that an average requirement of 1.5 mg/day has been established using allometric scaling based on the AR for non-pregnant women, considering an average increase in body weight during pregnancy of 12 kg; based on this, a recommended intake (PRI) of 1.9 mg/day was proposed. For lactating women, the AR is also increased in relation to non-lactating women due to the secretion of the vitamin into milk (0.291 mg/day) and an absorption efficiency of 95%, so that an AR of 1.65 mg/day and a PRI of 2 mg/day were set by the EFSA Panel for nursing mothers during the first 6 months of lactation. A summary of the DRVs established by the EFSA for riboflavin is shown in Table 1.

Milk and eggs provide unbound riboflavin, but most other foods primarily deliver FAD and FMN, which require a release from their carrier proteins. The stomach undergoes protein denaturation, and further hydrolysis takes place by alkaline phosphatases and FMN/FAD pyrophosphatases (Figure 3) in the brush border of the ileal enterocyte, yielding riboflavin that may be absorbed in the small intestine [51]. Three riboflavin membrane transporters have been identified, which are known as RFVT1 (formerly hRFT1), RFVT2 (hRFT3), and RFVT3 (hRFT2). The genes SLC52A1, SLC52A2, and SLC52A3 are responsible for encoding them in that order. The three transporters exhibit distinct subcellular localisations and tissue specificities [32]. Enterocytes take up riboflavin through a carrier-mediated process involving RFVT3. This process occurs at the apical membrane and is saturable. Studies have shown that this uptake process is linear up to approximately 30 mg of riboflavin per meal. Beyond this quantity, there is minimal additional absorption of riboflavin. Riboflavin may be released into the portal circulation and carried to the liver in its free form or as FMN. RFTV1 and RFVT2, located in the basolateral membrane of enterocytes (Figure 3), facilitate this movement [47]. Further riboflavin circulates in plasma by binding to albumin or immunoglobulins or transforming into its coenzyme forms in erythrocytes or leukocytes. The median plasma concentrations of riboflavin, FMN, and FAD in healthy adults have been estimated to be 10.5, 6.6, and 74 nmol/L, respectively [44].

After taking it in, cells quickly convert riboflavin into its biologically active cofactors with the aid of riboflavin kinase (RFK) (EC 2.7.1.26) and FAD synthase (FADS) (EC 2.7.7.2) [47]. RFK is an essential enzyme widespread in the body that transfers a phosphoryl group from ATP to riboflavin to produce flavin mononucleotide (FMN) [52], while FADS is responsible for FMN adenylation to FAD. RFK contains a six-stranded antiparallel beta barrel central core, which interacts with riboflavin and ADP [32]. Furthermore, by binding to tumour necrosis factor receptor 1 (TNFR1), RFK helps TNFR1 to physically and functionally couple to NADPH oxidase, a critical step for NADPH oxidase activation [31].

FADS synthase is responsible for FMN adenylation to FAD [53]. Alternative splicing of the FLAD1 gene generates different forms of FAD synthase, which are distributed across various parts of human cells [47,54,55]. It has been demonstrated that the most common form of this enzyme, cytosolic isoform 2, can function as both an FAD synthase and a hydrolase. The enzyme comprises an MPTb domain at the N-terminus that breaks down FAD, and a PAPS reductase domain at the C-terminus. The latter domain can catalyse FAD synthesis and has been renamed the FADS domain [56]. A variant of the FADS6 gene, made up of the PAPS reductase domain, was found in people with FLAD1 frameshift mutations. A functional analysis showed that FADS6 might act as an “emergency” enzyme, making it possible for people with biallelic FLAD1 frameshift variations to make cytosolic FAD [57]. This ability allows afflicted patients to survive [54]. Anticipating the absence of a mitochondrial-targeting peptide, FADS6 is expected to exist solely in the cytosol. Its job may include controlling the FAD transporter’s direction of travel by making it easier for FAD to enter the cell to help make flavoproteins [58].

SLC25A32 is a transporter located in the inner mitochondrial membrane, whose function is to import FAD from the cytosol into the mitochondria. FAD is essential for various mitochondrial enzymes, such as those involved in the respiratory chain and fatty acid beta-oxidation. SLC25A32 was initially discovered as a transporter of folate in mitochondria [54]. However, subsequent studies revealed it to be the human equivalent of the yeast mitochondrial FAD transporter FLX1. The protein FLX1 facilitates the transfer of the redox cofactor FAD across the mitochondrial membrane in both directions [56]. It remains uncertain if SLC25A32 regulates the FAD efflux from the mitochondrial matrix to the cytosol in human cells. Unbound flavins undergo rapid hydrolysis, releasing free riboflavin, which the body expels in urine. In general, it is assumed that riboflavin has low toxicity; therefore, supplementation has not been linked to any known problems, even when administered in high amounts, as often used in pharmaceutical treatments [31]. Because the body does not store riboflavin, any extra that is taken in beyond what the tissues need or what the kidneys can reabsorb is flushed out in the urine as riboflavin or metabolites, such as lumiflavin, 7-alpha-hydroxy riboflavin, and 10-hydroxyethylflavin [47]. Diet is the primary element influencing human riboflavin status on the general population. Although, in particular cases, other factors, such as pregnancy, physical activity, age, infections, and genetic abnormalities, may also affect riboflavin status.

## 5. Metabolic Role of Riboflavin

Respiratory complex I, also known as NADH dehydrogenase (EC 7.1.1.2), and complex II, also known as succinate dehydrogenase (EC 1.3.5.1), play a crucial role in energy generation in the mitochondrial respiratory chain. They are found in the inner mitochondrial membrane in eukaryotes and translocate electrons across it, generating the electrochemical gradient required for ATP synthesis [59]. Complex I requires FMN, whereas complex II requires FAD; both cofactors can accept and give one or two electrons through the tricyclic heteroaromatic isoalloxazine ring, being able to participate in a wide range of oxidation and reduction reactions. FAD is also a coenzyme for a range of dehydrogenases, hydrolases, oxidases, and transferases that participate in additional redox reactions of intermediary metabolism. These reactions include the initial stage of fatty acid β-oxidation, the oxidative decarboxylation of pyruvate and α-ketoglutarate, choline catabolism, purine catabolism, sphingosine synthesis, and the synthesis of cholesterol and steroid hormones. FAD has also a crucial role as a coenzyme for many cytochrome P-450 components, making it essential for the metabolism of medicines and different xenobiotics [60].

Riboflavin participates in the antioxidant defence system by activating the FAD-dependent enzyme glutathione reductase [54], which restores reduced glutathione from its oxidised state. Glutathione reductase is crucial in the glutathione redox cycle by regulating reduced glutathione levels, which helps safeguard live cells from the harmful impact of reactive oxygen species (ROS). The administration of riboflavin in animal models of chronic degenerative diseases associated with ROS, such as diabetes, renal toxicity, and hepatotoxicity [61], resulted in elevated levels of antioxidant enzymes, including glutathione reductase, and reduced glutathione, indicating that this vitamin has an impact on the antioxidant status. Aside from its crucial function in the glutathione redox cycle, riboflavin also indirectly safeguards against ROS by serving as a coenzyme for xanthine oxidase [62]. This enzyme facilitates the conversion of hypoxanthine and xanthine into uric acid, which is known to be a highly effective water-soluble antioxidant. Studies have shown that a riboflavin shortage leads to a lower activity of xanthine oxidase and a decrease in blood uric acid levels [63]. Riboflavin might also produce its effects by modifying the expression of genes and pathways involved in the activity of antioxidant enzymes [59].

Although riboflavin is recognised for its antioxidant properties, it can also act as a prooxidant when exposed to UVA radiation. The isoalloxazine ring of riboflavin absorbs energy from UVA radiation through photosensitisation, leading to the formation of singlet oxygen and other reactive oxygen species, which have the potential to cause damage to nearby molecules [64]. Therefore, riboflavin can potentially harm tissues exposed to high-intensity light, such as the skin and eyes, although other antioxidants, including ascorbate, carotenoids, tocopherols, and polyphenols, might help neutralise the negative impact of riboflavin when exposed to light [65]. On the other hand, the light-sensitive characteristics of riboflavin might be of interest for its use as an antibacterial agent and in the treatment of illnesses such as keratoconus and cancer [15]. Riboflavin is also known to act as a regulator of cryptochromes, which are light-sensitive flavoproteins found in the retina ganglion cells that are crucial for synchronising the circadian rhythms in response to daylight. However, a study on the molecular foundation of different animal cryptochromes revealed that those found in vertebrates do not possess the necessary structural characteristics to bind the photoactive flavin effectively, suggesting that riboflavin may have lost its functional significance in circadian photoreception in vertebrate animals throughout evolution [66]. Additional research is still required to elucidate the function of riboflavin in circadian biology.

As discussed below, riboflavin coenzymes participate in the metabolism of other B-type vitamins, such as folate, vitamin B12, vitamin B6, and niacin, as well as of iron, so that insufficient levels of riboflavin may disrupt their metabolic processes resulting in functional shortages of those nutrients.

## 6. Disorders in Riboflavin Metabolism: Malfunctions in Transportation

Brown–Vialetto-Van Laere syndrome is a rare autosomal recessive neurological disorder that results from insufficient levels of membrane riboflavin transporters (RFVTs), caused by mutations in the SLC52A2 (OMIM #61470) and SLC52A3 (OMIM #211530) genes [67]. Type 1 riboflavin transporter deficiency neuronopathy is a specific form of this condition caused by mutations in the SLC52A3 gene. It is known as Brown–Vialetto–Van Laere syndrome-1 (BVVLS1), consisting of a progressive neurological disorder characterised by bulbar palsy and sensorineural deafness [68]. Fazio–Londe disease is a disorder related to BVVLS1 that does not involve sensorineural deafness [69]. Mutations in the SLC52A2 gene lead to type 2 riboflavin transporter deficient neuronopathy, known as Brown–Vialetto–Van Laere syndrome-2 (BVVLS2) [68]. Deficiency in riboflavin transporter 1 is another quite rare metabolic disorder due to autosomal dominant pathogenic variants in the SLC52A1 gene (OMIM #615026). Only a few cases of this pathology have been reported worldwide in newborns, leading to severe symptoms consistent with Multiple Acyl-CoA Dehydrogenase Deficiency (MADD) [31]. This condition has been associated with maternal riboflavin deficiency, and supplementation with oral riboflavin led to the resolution of the clinical symptoms [70]

A mitochondrial FAD transporter deficit has been reported in two cases of individuals who had mutations in both copies of the SLC25A32 gene (OMIM #616839), which encodes the mitochondrial FAD transporter. The first case was a 14-year-old female who showed recurrent exercise intolerance with biochemical characteristics of MADD without having mutations in the genes related to that disorder [71]. The second case was a 51-year-old patient with severe neuromuscular symptoms accompanied with speaking and swallowing difficulties, following a progressive impairment in motor abilities since the age of 3 years [72]. The muscle biopsy showed the presence of ragged-red fibres, lipid storage, and fibres with reduced staining for succinate dehydrogenase (SDH) and cytochrome c oxidase (COX) in both patients. SDH is an FAD-dependent enzyme part of the mitochondrial respiratory chain complex II. At the same time, COX is part of the mitochondrial respiratory chain complex IV, and complex II deficiency was also observed in the second patient’s muscle tissue and the first patient’s cultured skin fibroblasts [71,72]. After receiving oral riboflavin supplementation, both patients significantly improved the clinical and biochemical manifestations, including increased exercise tolerance and endurance.

## 7. Primary Dysfunction in Riboflavin Coenzyme Pathways

The main issue in riboflavin coenzyme metabolism is a deficiency in FAD synthase. It has been shown that mutations in the FLAD1 gene, encoding FADS, can cause a neuromuscular disease. Lipid storage myopathy, metabolic abnormalities resembling MADD, and deficiencies in numerous respiratory chain enzymes are the hallmarks of this disorder, which is treatable. Thirteen reports had been collected in the literature, as reviewed by Olsen et al. [71]. In eleven instances (85%), the onset occurred during infancy; the common symptoms in almost all those newborns were hypotonia and profound muscular weakness, resulting in difficulty with feeding (such as poor suck and swallow) and inadequate respiratory function. The adult-onset instances exhibited symptoms of exercise intolerance and gradual muscular weakening, accompanied by problems in walking, bilateral foot drop, and weakness in the arms in the older patient. The FADS absence is associated with a distinct muscle pathophysiology characterised by significant lipid accumulation and a widespread reduction in COX and SDH histochemical staining. In skeletal muscle biopsies, respiratory chain enzyme deficits were detected, which affected respiratory chain complexes I, II, III, and IV to varying degrees [72].

Three distinct homozygous FLAD1 mutations have been described in individuals diagnosed with FADS insufficiency. One mutation seems to be widespread, indicating a potential founder impact. It consists of a four-base pair deletion (c.401_404delTTCT, first reported as c.397_400delTTCT) in the MPTb domain, which causes a frameshift and premature termination of the protein (Phe134CysfsTer8) [73]. It was reported in five children from four families; all of them perished within a time frame of 4 to 8 months after birth [55,73,74]. In the report, three out of four individuals with a mutation in at least one FADS domain that affected a single amino acid lived for a long time (between 22 and 56 years), whereas the fourth infant died after just three days of life [55]. In contrast, eight patients showing biallelic frameshift mutations in the MPTb domain passed away, with most of them dying during infancy [55]. One, diagnosed with MADD via prenatal screening, received riboflavin treatment for the first 29 months of their life; however, starting from the age of 3, progressive myopathy was developed, and, at the age of 8, a homozygous nonsense mutation in the MPTb domain of FLAD1 was found, which produced a shortened protein that retained some level of FADS activity [75]. In seven of eight MADD-diagnosed patients, despite the premature death, supplementing with riboflavin led to positive clinical outcomes that included a significant reduction in muscle symptoms and increased muscle strength [75,76]. In the one that reached adult age, nutritional treatment consisting of a high-carbohydrate, moderate-protein, and low-fat (maximum 20 g fat per day) diet helped improve the symptoms and correct the biochemical abnormalities. Administering riboflavin to two siblings with the c.401_404delTTCT mutation in FLAD1 since the age of three months resulted in slight positive effects on their spontaneous activity, muscle tone, vomiting, and alertness. However, it was not able to halt the progression of the disease, as both infants passed away before reaching six months of age [74]. There is some suggestion that biallelic frameshift mutations in exon 2 of FLAD1 could show some response to riboflavin supplementation. In fact, early treatment with riboflavin was reported to have reduced the severity of symptoms in an 8-year-old child with a homozygous nonsense mutation in FLAD1, that showed aberrant biochemical results in the newborn screening test for MADD [75]. In general, for individuals with FADS deficiency, it is advisable to administer a substantial dose of riboflavin. When suspecting the diagnosis, it is recommended that we initiate riboflavin supplementation while awaiting mutational investigation. If the diagnosis is not confirmed, the patient should stop taking supplements. An additional inquiry is warranted to explore the possibility of using large doses of riboflavin to prevent or improve illness symptoms in individuals before or after birth [74]. The employment of some mono-functional isoforms of FAD synthase, such as FADS6, has been suggested as an approach for therapy intervention in individuals with FADS defects that do not respond to riboflavin treatment. This isoform, found in patients with Riboflavin-Responsive MADD, might explain why individuals with biallelic FLAD1 frameshift variants still hold substantial FADS activity and, thus, survive. The over-expression of human FADS6 in *Escherichia coli* allowed its production and functional characterisation opening prospects for its bacterial synthesis and therapeutical use [58].

No cases of riboflavin kinase (RFK) deficiency have been reported in humans. However, researchers have demonstrated that the production of FAD, a cofactor essential for various cellular processes, relies on RFK. Given that RFK is considered the rate-limiting step in this pathway, its deficiency may be incompatible with life. Studies on mice, where the RFK gene was intentionally deactivated, resulted in embryo death before day 7.5 of gestation, supporting the notion that RFK deficiency could be fatal [31]. Nevertheless, hypomorphic RFK mutations might lead to a clinical manifestation like that in individuals with FLAD1 mutations. In the future, whole-exome and -genome sequencing might help find people who have not been diagnosed yet but have lipid storage myopathies and symptoms like MADD.

## 8. Malfunctions within the Human Flavoproteome

The human flavoproteome consists of at least 90 different proteins, each with various functions [77]. Approximately half of these proteins have been linked to human diseases that exhibit significant clinical heterogeneity, such as Leigh Syndrome and other conditions related to the mitochondrial respiratory chain, as well as disorders associated with mitochondrial and peroxisomal fatty acid oxidation [78]. Other diseases include pyridoxal phosphate-responsive epilepsy, trimethylaminuria, rhizomelic chondrodysplasia punctata, porphyria variegata, chronic granulomatous disease, and defects in steroidogenesis and bile acid synthesis. There is a lack of information on the effectiveness of riboflavin treatment and whether high dosages could help overcome several of these problems.

Electron-transfer flavoprotein dehydrogenase (ETFDH) deficiency or one of the two electron-transfer flavoproteins (ETFA and ETFB) causes MADD. These proteins transfer electrons from acyl-CoA dehydrogenases to coenzyme Q10 in the mitochondrial respiratory chain. MADD has two clinical presentations, a severe neonatal onset multisystemic form, and a less severe myopathic form that appears later in life. This latter often accompanies exercise intolerance, respiratory insufficiency, or rhabdomyolysis and can be treated with riboflavin [79]. In some cases, where there may also be a secondary deficit of coenzyme Q10, riboflavin supplementation has been effective in improving clinical symptoms and metabolic abnormalities in over 95% of individuals with late-onset MADD [80]. Most of these patients (93%) had ETFDH mutations.

ACAD9 is an enzyme that plays a primary role in the formation of respiratory complex I and also has an extra function in the oxidation of fatty acids [81]. ACAD9 deficiency manifests with various clinical features, including early-onset cardiac problems, such as electrical hypertrophy and severe hypertrophic, dilated, or combined cardiomyopathy. Other symptoms encompass exercise intolerance, myopathy, lactic acidosis, and neurological abnormalities such as stroke-like episodes, ataxic gait, bradykinesia, and bradylalia. ACAD9 mutations are the primary reason for hypertrophic cardiomyopathy with isolated complex I deficit [82]. The study by Nouws et al. [81] showed that riboflavin directly increased the amounts of ACAD9 protein, positively impacts the assembly of complex I, and can act as a chemical chaperone by improving the folding of specific mutant ACAD9 proteins. In a study over 70 patients, Repp et al. [83] reported that riboflavin supplementation improved the condition of 65% of individuals with ACAD9 deficiency.

Dihydrolipoamide dehydrogenase, also referred to as E3 and encoded by the DLD gene, is a flavoprotein that is present in three mitochondrial α-ketoacid dehydrogenase multi-enzyme complexes: pyruvate dehydrogenase complex (PDHC), α-ketoglutarate dehydrogenase complex (KGDC), and branched-chain α-keto acid dehydrogenase complex (BCKDC). The range of observable characteristics of DLD deficiency is diverse. It includes conditions such as early-onset encephalopathy with progressive muscle weakness, failure to grow, low blood sugar [84], ketoacidosis, and encephalopathy. It can also manifest as Leigh syndrome or a recurring presentation like Reye syndrome, where the individual has normal intelligence and no lasting neurological issues between episodes of metabolic disturbance [85], but reported a myopathic phenotype sensitive to riboflavin [85]. This phenotype exhibits exertional tiredness, intermittent increases in blood lactate, ketoacidosis, the elevation of creatine kinase, and mitochondrial proliferation. Giving extra riboflavin eliminates all muscle weakness, fixed metabolic problems, helped the DLD protein partially recover, and lowered the production of reactive oxygen species (ROS) in fibroblasts [85]. These findings support the idea that riboflavin has a chaperone-like effect, promoting the stability and proper folding of the DLD protein [86].

The AIFM1 gene on the X chromosome produces an apoptosis-inducing factor (AIF) protein. The flavoprotein AIF is found in mitochondria and has two jobs: a death effector that kills cells without the help of caspases, and an FAD-dependent NADH oxidoreductase that helps with redox control and oxidative phosphorylation. Cell death occurs when AIF moves from the mitochondria to the nucleus in response to apoptotic signals. AIFM1 mutations link to various clinical phenotypes, including a severe form of mitochondrial encephalomyopathy characterised by combined oxidative phosphorylation deficiency (OMIM #300816), as well as Cowchock syndrome, an X-linked Charcot–Marie–Tooth disease characterised by axonal sensorimotor neuropathy, sensorineural deafness, and cognitive impairment (CMTX4, OMIM #310490). A recent study pointed to AIFM1 mutations as the cause of cerebellar ataxia, which exhibits partial responsiveness to riboflavin treatment [87].

## 9. Riboflavin’s Interactions with Other Nutrients

Riboflavin coenzymes participate in the metabolism of folate, vitamin B12, vitamin B6, and niacin. FAD is essential for the activity of methylenetetrahydrofolate reductase (MTHFR), an important enzyme in folate metabolism, responsible for converting 5,10-methylenetetrahydrofolate into 5-methyltetrahydrofolate [68]. The enzyme methionine synthase reductase (MTRR) also depends on FMN and FAD coenzymes. MTRR helps produce methylcobalamin, the physiologically active form of vitamin B12 required by methionine synthase (MS) [88]. One-carbon metabolism relies on MTHFR and MS, which convert homocysteine to methionine through remethylation. ATP then activates methionine to produce S-adenosylmethionine (SAM), a universal methyl donor that is essential for various substances, including DNA, proteins, phospholipids, neurotransmitters, and hormones [89].

Furthermore, FMN is a cofactor for pyridoxine (pyridoxamine) 5′-phosphate oxidase (PNPO; EC 1.4.3.5), an essential enzyme in vitamin B6 metabolism. PNPO converts the 5-phosphates of pyridoxine and pyridoxamine into pyridoxal-5′-phosphate (PLP), the biologically active form of vitamin B6. PLP is necessary for various reactions involved in amino acid metabolism, one-carbon metabolism, glycogenolysis, and gluconeogenesis. Pyridoxal kinase can also phosphorylate pyridoxal to produce PLP through pyridoxal phosphorylation, but PNPO has a higher rate of PLP generation, making it more efficient. Consequently, riboflavin is crucial for maintaining sufficient levels of vitamin B6 [90]. Another pyridoxal phosphate-dependent enzyme is kynurenine 3-hydroxylase (K3H), a flavoprotein involved in the tryptophan pathway, making its conversion to niacin possible [91]. Thus, riboflavin deficiency may contribute to the etiology of pellagra when intakes of tryptophan and niacin are insufficient.

Riboflavin also interacts with iron. Unbound reduced riboflavin, which is not associated with proteins, plays a role in releasing iron from ferritin, a storage protein, in the cytoplasm. Both FMNH2 and FADH2 are involved in electron transfer pathways leading to a reduction in Fe^3+^ to Fe^2+^, promoting the crucial process of iron mobilisation from ferritin [92]. Ascorbate and glutathione are other agents that may release Fe^2+^ cations from ferritin, even though the reduced forms of riboflavin are more efficient in this action [93]. Extracting iron from ferritin is significant for all tissues, but it is particularly crucial for the gastrointestinal mucosa due to its potential impact on iron absorption. Animal and in vitro mechanistic investigations indicate that the level of riboflavin may affect both the absorption of iron in the gastrointestinal tract and the loss of iron [72]. Human studies utilising an iron stable isotope found no significant alterations in iron absorption following riboflavin supplementation [94], although they noted improvements in various haematological parameters among participants, suggesting that the release of naturally occurring iron from ferritin likely caused the effect [55].

Functional deficiencies of other micronutrients may occur because of riboflavin deficiency. The decreased levels of haemoglobin and the presence of hypochromic anaemia in riboflavin deficiency mainly occur due to the hindered release of iron from cells, namely, the liver’s ferritin [86], which necessitates the reduced forms of riboflavin, as indicated above. Research conducted on population groups with inadequate or below-optimal levels of riboflavin, such as school children, men, pregnant women, lactating women, and women at reproductive age, has demonstrated that supplementing with riboflavin improves their haematological status. Similarly, an intervention study conducted on elderly individuals with insufficient vitamin levels initially showed that supplementing with riboflavin not only improved riboflavin levels but also enhanced plasma PLP, the active form of vitamin B6 [86]. Empirical data strongly support previous human investigations indicating that riboflavin supplementation increases the activity of FMN-dependent PNPO in erythrocytes, required to produce PLP [95].

Riboflavin insufficiency hinders folate metabolism, particularly in persons with two copies of the common C677T mutation in MTHFR, resulting in significant functional and health implications. Individuals with the MTHFR 677TT genotype have a higher likelihood of developing hypertension. However, taking riboflavin, the MTHFR cofactor, may reduce blood pressure, regardless of whether antihypertensive drugs or other specific kinds of medication are used [93]. Molecular studies indicate that, in those afflicted, there is a higher likelihood for the riboflavin cofactor (FAD) to separate from the enzyme’s active site, causing it to become inactive, resulting in poor folate metabolism [96]. Moreover, a riboflavin deficiency is linked to increased levels of homocysteine, while providing riboflavin supplements to individuals with the 677TT genotype significantly reduces homocysteine levels, suggesting that sufficient riboflavin levels can restore MTHFR activity [97]. Genome-wide association studies and clinical investigations have established a link between the MTHFR C677T polymorphism and a heightened susceptibility to hypertension and hypertension in pregnancy, with a potential increase in risk of up to 87% [98]. The centre’s randomised trials have shown that riboflavin can lower systolic blood pressure by 6 to 13 mm Hg in hypertensive individuals with the MTHFR 677TT genotype. The discovery of riboflavin’s new involvement in regulating blood pressure in a genotype-specific class might have significant ramifications for public health. The potential to mitigate or cure hypertension in certain groups around the globe is significant, given that this genetic variation impacts 10% of the global population on average; while its prevalence varies between 4–26% in Europeans, higher rates are found in the southern regions. In Northern China, the prevalence is 20%, while, in Mexico, it could be as high as 32% [99].

Riboflavin deficiency might also lead to other health issues. Animal studies have pointed out that riboflavin inadequacy can increase the risk of cancer, although the epidemiological data, including meta-analyses, on the association with human colorectal cancer and breast cancer are inconsistent [31]. Phototherapy, which uses blue light to treat newborn hyperbilirubinemia, has been shown to cause the breakdown of riboflavin and a shortage of the vitamin [100]. Using riboflavin supplementation in this situation is not recommended due to the potential DNA damage caused by the byproducts of riboflavin photolysis. On the other hand, studies in areas where malaria is prevalent revealed that people who have insufficient riboflavin are less susceptible to malaria and have lower parasitemia levels. However, those individuals may experience a more severe progression of the illness than those with adequate riboflavin levels [31].

## 10. Implications of Riboflavin in Diseases

### 10.1. Deficiency Signs

**Figure 4:**
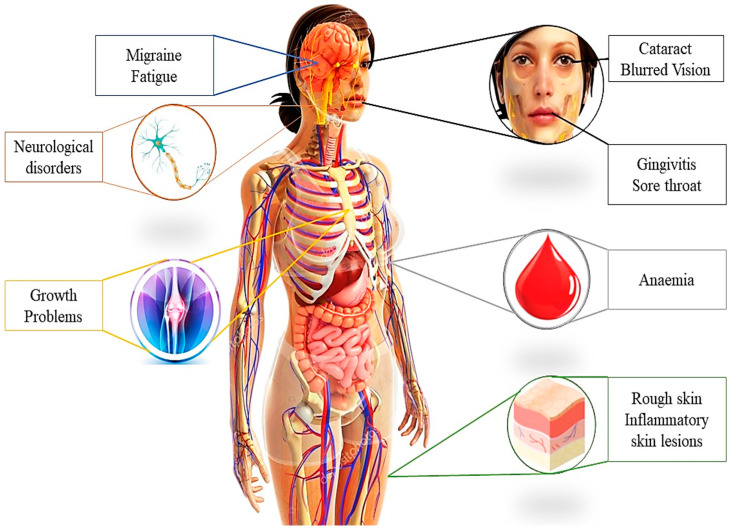
Main signs and symptoms of riboflavin deficiency. Figure adapted from [46].

The signs of riboflavin deficiency are known as ariboflavinosis. They include cheilosis (inflammation and cracking of the lips), angular stomatitis (inflammation and cracking at the corners of the mouth), glossitis (inflammation of the tongue), redness and swelling in the mouth and throat, and severe anaemia with less red blood cell production (erythroid hypoplasia) (Figure 4) [31]. Additional symptoms may include the growth of blood vessels in the eye, inflammation of the skin (seborrhoeic dermatitis), and changes in the nervous system. Because riboflavin interacts with other nutrients, these symptoms are not specific to ariboflavinosis and may also result from other vitamin deficiencies. The clinical manifestations of riboflavin insufficiency in humans become evident after a few months of consuming less than 0.5–0.6 mg/day of vitamin B2 [18]. The main signs and symptoms of riboflavin deficiency, along with their correlation with various pathologies, will be discussed in the following sections.

### 10.2. Anaemia

Anaemia occurs due to insufficient red blood cells or haemoglobin, resulting in a decrease in oxygen-carrying capacity. Riboflavin contributes to erythropoiesis, enhances iron absorption, and assists in releasing ferritin from tissues [101]. A shortage in riboflavin can lead to a notable increase in the loss of iron via the gastrointestinal tract and a reduction in the release of iron from its body storages [102].

The Jiangsu Nutrition Study conducted in China found that insufficient riboflavin intake increases the risk of developing long-lasting anaemia, with women under the age of 50 being particularly vulnerable [103]. Generally, cross-sectional studies show a negative association between riboflavin intake and anaemia; however, there is a limited availability of prospective population research on this topic. [104]. If there is insufficient riboflavin intake, iron supplementation may not be the most effective method for preventing anaemia in the population. In 1973, a study conducted in the United Kingdom demonstrated that riboflavin, when used alone without supplementary iron, could improve the haematologic status of young women [104]. Therefore, preventing anaemia by addressing riboflavin insufficiency becomes a crucial aspect, which necessitates assessment and intervention studies on a population-based level [104]. Further investigation is, therefore, needed to determine the precise effect of riboflavin on iron absorption and the extent of iron lost due to a deficiency of the vitamin.

### 10.3. Migraine

Currently, there is not a comprehensive theory or hypothesis explaining all phenomena associated with migraines. A frequently cited explanation for the development of migraines is that a malfunction in the mitochondria, which results in a disruption in the processing of oxygen, may contribute to their onset [105]. In this respect, riboflavin could have the potential to effectively prevent migraines by reversing the dysfunction [105]. Riboflavin helps the mitochondrial electron transport chain start and is also a part of the Krebs cycle, giving energy [34]. Thus, increasing riboflavin availability might improve the brain mitochondria’s functioning and help prevent migraines [34]. Earlier studies have shown that riboflavin decreases the occurrence of migraine episodes and the number of days with headaches reported, while administering magnesium did not show any discernible difference between the experimental and placebo groups [106]. Riboflavin is considered safe, well-tolerated, and affordable [105], although there seem to be variations in the response to it depending on gender and age. A retrospective study conducted by Condo et al. [107] found that prophylactic-treatment riboflavin could be efficient for reducing the incidence of migraines in children and adolescents, as well as for decreasing the severity of the symptoms, especially in male patients.

### 10.4. Diabetes Mellitus

Oxidative stress is thought to play a significant role in the development of type-2 diabetes mellitus (T2DM) [16]. Studies have shown that adding riboflavin to the diet of diabetic mice improved markers of both renal and liver function. Untreated mice exhibited significant damage to the microstructure of the liver, including changes in the shape of hepatocytes, a low presence of cell organelles, and poorly maintained hepatic cords. The renal tubule and glomeruli were also significantly impacted. The treatment with riboflavin significantly decreased these alterations, suggesting that riboflavin therapy could help reduce the likelihood of diabetes complications by decreasing oxidative-stress-induced inflammation [16].

### 10.5. Cataracts

Riboflavin could have a crucial effect in preventing the development of cataracts. The eye contains high quantities of glutathione that, in its reduced form (GSH), protects the lens proteins from oxidative damage, preventing lens optical cloudiness. Thus, a decrease in GSH levels is associated with the development of cataracts. As above commented, riboflavin serves as a cofactor for glutathione reductase (GR), the enzyme responsible for converting oxidised glutathione (GSSG) into GSH, so that riboflavin insufficiency increases their risk of developing cataracts in the elderly [107].

An early comprehensive randomised, double-blind, controlled trial conducted in China examined the effects of multivitamin/mineral supplements over a period of 5 to 6 years on cataract development in individuals aged 45 to 74 years. The study found a statistically significant reduction in the prevalence of nuclear cataracts among those who took a daily dose of 3 mg of riboflavin and 940 mg of niacin. The greatest benefit was observed in the group aged 65 to 74 years, with up to a 44% reduction in prevalence [108].

As an antioxidant, riboflavin can play a role in reducing oxidative stress, either acting independently by converting reduced riboflavin to its oxidised form or as part of the glutathione redox cycle. Specifically, it can help prevent lipid peroxidation and oxidative harm caused by reperfusion. Additionally, it neutralises reactive oxygen species and peroxides, including hydroperoxides. Recent research has shown that riboflavin may efficiently permeate the corneal stroma by passing through the endothelium after intracameral injection [104]. A comparable enrichment effect can be produced after applying riboflavin to a corneal surface stripped of its epithelium [108,109].

All in all, riboflavin is crucial in maintaining the antioxidant state of cell systems, both independently and as a component of the glutathione reductase and xanthine oxidase system. Furthermore, it is also capable of modulating supplementary antioxidant enzymes, such as superoxide dismutase (SOD), catalase, and glutathione peroxidase. Thus, either a lack of riboflavin or its addition via supplementation impacts lipid peroxidation, causing detrimental or beneficial effects, respectively [109].

### 10.6. Childhood Neuropathy

Mutations in the SLC52A2 gene, which encodes the riboflavin transporter RFVT2 and is responsible for Brown–Vialetto–Van Laere syndrome, decrease riboflavin absorption and have been related with neurological disorders in early stages of development, as discussed above. It has been reported that patients with these mutations show significant and long-lasting clinical and biochemical improvements in response to high-dose oral riboflavin therapy, counteracting the advancement of this neurodegenerative illness [97].

### 10.7. Hypertension

Hypertension is one of the primary causes of death globally, resulting in about 8 million premature deaths per year [110]. The precise underlying mechanisms of hypertension are yet to be understood, but several lifestyle and genetic risk factors have been identified. Genome-wide association studies have found several genetic loci related to blood pressure variation [111]. An example of such a location is near the gene that encodes methylenetetrahydrofolate (MTHFR), the enzyme responsible for metabolising folate, necessary for producing 5-MTHFR. It has been proposed that riboflavin could be involved in regulating blood pressure, particularly in patients with the MTHFR 677TT genotype [111]. As previously discussed, riboflavin serves as a cofactor (FAD) for MTHFR, and the reduced activity of this enzyme in persons with the TT genotype is due to a higher likelihood of FAD loss. Riboflavin supplementation helps counteract the increased risk of FAD loss from the active site and reduces homocysteine levels and blood pressure in individuals with the TT genotype, compared to those with CC or CT genotypes. Supplementing with riboflavin may also stabilise the variant MTHFR enzyme, potentially restoring 5-MTHF levels in vascular cells. This restoration could enhance nitric oxide availability, improve endothelial function, and reduce blood pressure [111].

### 10.8. Cancer

Cancer is a medical condition originating from the uncontrolled multiplication and spread of abnormal cells in the body. Multiple investigations have shown a correlation between riboflavin deficiency and the suppression of some tumour development in experimental animals and potentially in humans [112]. However, the exact processes that cause this phenomenon are yet to be explained. Riboflavin has received less attention than other factors in relation to cancer, although there is growing interest due to flavin’s recognised involvement in folate metabolism and the potential synergistic protective impact of these two vitamins [6]. Folate is crucial in essential processes such as DNA synthesis, repair, and methylation, mechanisms fundamental for explaining how folate may potentially prevent cancer. The common thermolabile variation of the MTHFR genotype can affect the function of folate. Homozygosity for this variant leads to reduced enzyme activity, lower levels of folate in the plasma and red blood cells, and increased plasma homocysteine levels. As previously indicated, in the form of FAD, riboflavin acts as a cofactor for MTHFR, which provides evidence for specific interactions between riboflavin status, folate status, and genotype in determining plasma homocysteine levels, which is a functional measure of folate status. The MTHFR C677T polymorphism interacts with folate and riboflavin in influencing the risk of cancer, and this interaction differs depending on the specific location of the cancer.

Many data suggest that this genetic variation has a protective effect against the development of colorectal cancer. However, its impact on the risk of cervical cancer remains uncertain [113]. The study investigated the plasma levels of a riboflavin carrier protein (RCP) stimulated by oestrogen in breast cancer patients [114]. It was found that a serum RCP level of more than 1.0 ng/mL was strongly associated with the occurrence of breast cancer. This finding suggests that RCP levels might serve as valuable diagnostic markers for breast cancer, even in its early stages [6]. Epidemiological studies have also identified a connection between esophageal cancer and diets deficient in riboflavin [61], although other studies have not confirmed this correlation [61]. Insufficient levels of riboflavin have also been indicated as a contributing factor for cervical dysplasia, a preliminary state for invasive cervical cancer [6] in patients with non-small-cell lung cancer at various stages [6]. The reduced concentrations of vitamin B2 and B6 in red blood cells, which are inversely associated with plasma ghrelin, further emphasise the significance of these vitamins in individuals diagnosed with lung cancer [115]. More research is, however, required to elucidate the mechanism by which vitamins and other micronutrients might contribute to the prevention and treatment of cancer.

## 11. Measurable Indicators of Riboflavin Concentrations

This section provides a comprehensive guide on determining riboflavin levels, a crucial step in assessing nutritional status and identifying potential health complications. It discusses measurable indicators for riboflavin levels, providing insights for researchers and healthcare professionals. The discussion covers traditional serum markers and innovative techniques used in clinical practice and research settings. The section aims to contribute to understanding nutritional assessment and intervention strategies for optimal health outcomes by highlighting the significance of riboflavin levels.

### 11.1. Erythrocyte Glutathione Reductase Activation Coefficient

Erythrocyte glutathione reductase (EGR) is an enzyme present in red blood cells that helps reduce the disulfide link in oxidised glutathione (GSSG) to produce reduced glutathione (GSH) [76]. The measurement of EGR activity is widely used to determine riboflavin status. It can be determined spectrophotometrically by quantifying in vitro the oxidation of NADPH to NADP, before and after the addition of the FAD coenzyme. The EGR activation coefficient (EGRac), calculated as the ratio of enzyme activity with or without adding FAD, indicates the degree of tissue saturation with riboflavin. An EGRac value of 1.3 is the typically used cutoff to define riboflavin deficiency; the higher the EGRac value, the lower riboflavin status [116]. EGRac is considered a reliable biomarker for identifying severe riboflavin deficiency and monitoring the effectiveness of riboflavin replacement [117]. A strong correlation between dietary riboflavin intake and EGRac was found in the Adult Nutrition Survey (NANS) conducted on a cohort of 5612 Irish adults aged 18–102 years [117].

### 11.2. Direct Riboflavin Biomarkers

Few studies have directly assessed riboflavin status by measuring its levels in plasma and erythrocytes. Erythrocyte riboflavin is considered a reliable indicator of long-term riboflavin consumption with values significantly correlated with those obtained by the reference technique, EGRac.

Furthermore, direct riboflavin measurement by liquid chromatography coupled with tandem mass spectrometry has several advantages over EGRac, such as convenience and accessibility for population surveys [18]. It has been shown that individuals with clinical symptoms of riboflavin shortage have lower levels of erythrocyte riboflavin than healthy adults. Supplementing with a small amount of riboflavin for 12 weeks resulted in higher erythrocyte FMN and FAD [118]. Plasma biomarkers, such as riboflavin and FMN, have also shown promising outcomes and are responsive to low-dose riboflavin supplementation, while plasma FAD concentrations have only shown mild responses to intervention [17]. Further research is necessary to confirm the accuracy of these specific biomarkers. The absence of easily measurable biomarkers results in a need for more data on riboflavin levels worldwide, and concerns about a low riboflavin status are more prevalent than commonly acknowledged.

### 11.3. Excretion of Riboflavin in Urine

The excretion of riboflavin in urine is also regarded as a reliable measure of its short-term state. Dietary riboflavin intake can be quantified when the body’s tissues reach maximum saturation, by a change in the urine excretion curve in direct correlation with the amount of riboflavin consumed. Experimental research has shown that, when the intake of riboflavin rises, the rates of excretion of riboflavin in urine steadily increase to the point of tissue saturation.

Once the riboflavin intake surpasses that threshold, each further increase leads to a significant increase in the rate at which it is excreted [119]. Riboflavin concentrations in urine may be assessed in urine samples obtained during a period of fasting, at arbitrary intervals, or over a span of 24 h [111]. These measures may be conducted with or without considering the creatinine percentage. Deficiency was defined as a complete excretion of riboflavin in the urine during a 24 h period that is less than 40 mg/day (or 27 mg/g creatinine) [112]. Values ranging from 40 to 120 mg/day (or 80 mg/g creatinine) showed insufficiency, while values over 120 mg/day indicated adequacy in adults. Various factors may interfere with the elimination of riboflavin, including the use of oral contraceptive drugs, pregnancy, some antibiotics, and mental therapies [119]. Some methods can be used, such as fluorometric high-performance liquid, which provide advantages in terms of sensitivity, rapidity, and the absence of pretreatment procedures [44].

## 12. Conclusions and Final Remarks

Riboflavin is crucial in energy metabolism and significantly enhances the cellular antioxidant capacity. Additionally, it interacts metabolically with other essential nutrients such as iron, vitamin B6, and folate, underscoring its significance in maintaining health and preventing illnesses. The physiological activities of riboflavin are vital across all life stages, emphasising its necessity for overall well-being. A riboflavin deficiency can lead to several adverse health outcomes, including anaemia, dermatitis, and cataracts, with anaemia being a prominent example.

The global prevalence of riboflavin insufficiency, particularly during pregnancy, is a pressing concern that demands our immediate attention. Current research must focus on elucidating the relationship between riboflavin deficiency and anaemia in pregnant women and investigating its potential impact on hypertensive disorders during gestation. The extent of global riboflavin insufficiency may be more widespread than generally recognised, a fact that remains largely unnoticed in many countries due to the infrequent measurement of riboflavin biomarkers. This underscores the need for additional research and the potential impact it can have.

Accurately assessing riboflavin status in human populations is not just a scientific endeavour but a crucial step towards identifying deficiencies and implementing necessary public health interventions. The development and validation of accessible riboflavin biomarkers should be a priority, particularly for use in large-scale nutrition surveys. These biomarkers would not only facilitate a more comprehensive assessment of riboflavin status globally but also pave the way for targeted interventions, significantly contributing to the improvement in public health.

While dairy products are rich sources of riboflavin, other food sources often lack sufficient levels to meet average dietary requirements. Therefore, exploring interventions such as fortifying foods with riboflavin is crucial. Re-evaluating the recommended dietary intake of riboflavin is also necessary, given emerging scientific evidence highlighting previously unknown functional and health benefits of riboflavin within the typical dietary range. Some examples of riboflavin-rich foods include eggs, lean meats, and leafy green vegetables.

Future research should not just aim to provide a deeper understanding of riboflavin’s role in human health. The potential of riboflavin is vast and untapped, and it is up to us to explore it. Additionally, educating the public about the importance of riboflavin-rich diets and the potential benefits of fortified foods can help mitigate the risk of deficiency. Addressing riboflavin insufficiency is not just a step; it is a leap towards improving global health outcomes.

## Figures and Tables

**Table 1:** DRVs established by the EFSA for riboflavin (adapted from the EFSA Panel on Dietetic Products, Nutrition, and Allergies [49]).

Age	Average Requirement (mg/Day)	Population Reference Intake (mg/Day) ^a^
**7–11 months**	-	0.4 ^b^
**1–3 years**	0.5	0.6
**4–6 years**	0.6	0.7
**7–10 years**	0.8	1.0
**11–14 years**	1.1	1.4
**15–17 years**	1.4	1.6
**≥18 years**	1.3	1.6
**Pregnancy**	1.5	1.9
**Lactation**	1.7	2.0

^a^ The values for AR and PRI in this table have been rounded to the nearest tenth decimal place. ^b^ Adequate Intake.

## Data Availability

No new data were created or analyzed in this study. Data sharing is not applicable to this article.

## Supplementary Materials

The following supporting information can be downloaded at: https://www.mdpi.com/article/10.3390/foods13142255/s1, Figure S1. The Structure of Riboflavin.
