# Supplementary figures and images for: Revitalising Riboflavin: Unveiling Its Timeless Significance in Human Physiology and Health
Source: Foods. 2024 Jul 17;13(14):2255. doi: 10.3390/foods13142255 (PMC11276209; doi:10.3390/foods13142255)

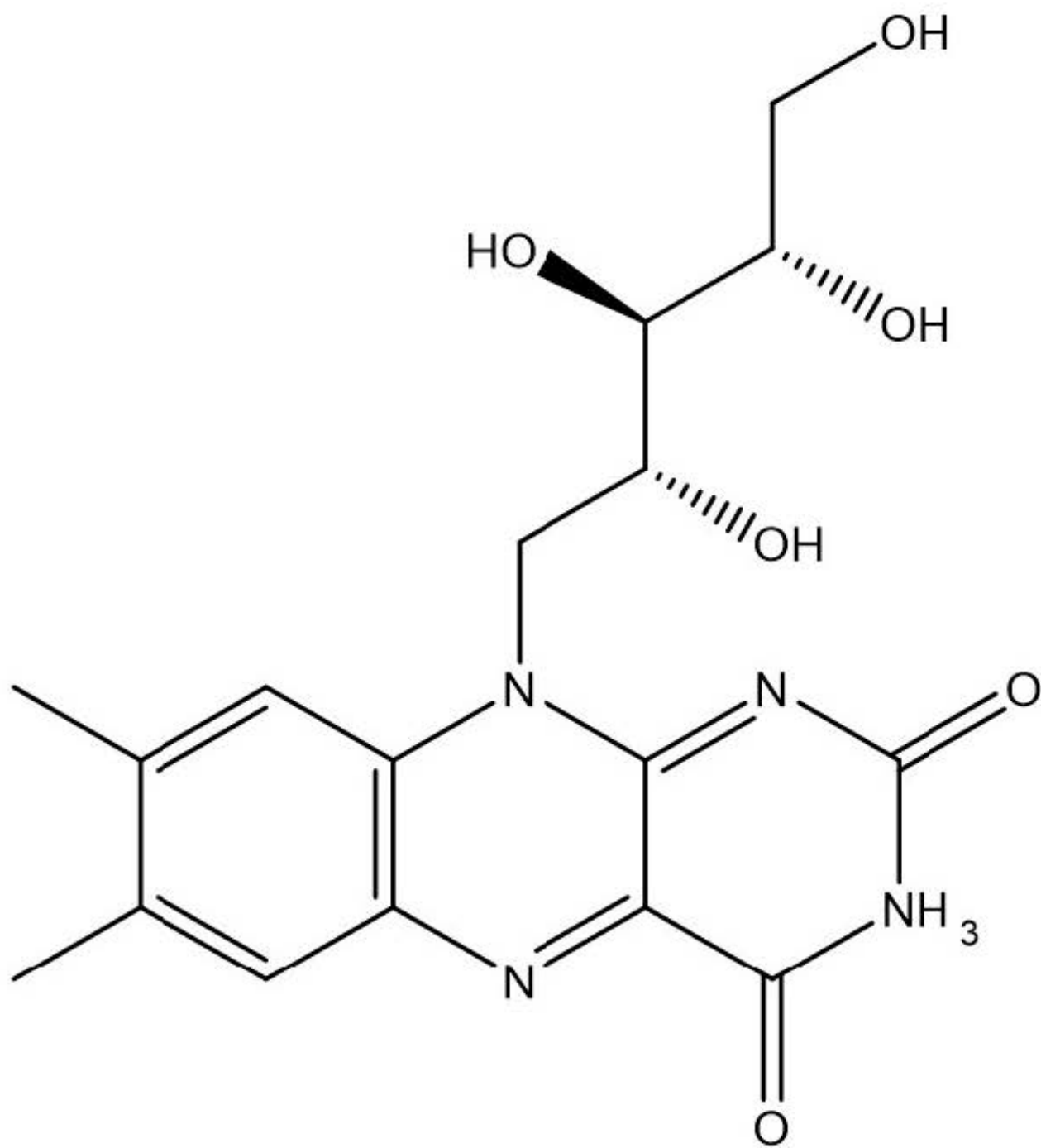

Figure S1. The Structure of Riboflavin

Supplement: Supplementary file 1 [file foods-13-02255-s001.zip › foods-3090759-supplementary.pdf]
